# Progress in adolescent sexual and reproductive health and rights globally between 1990 and 2016: what progress has been made, what contributed to this, and what are the implications for the future?

**DOI:** 10.1080/26410397.2020.1741495

**Published:** 2020-04-07

**Authors:** Venkatraman Chandra-Mouli, Elsie Akwara, Danielle Engel, Marina Plessons, Mengistu Asnake, Sunil Mehra, Bruce Dick, Jane Ferguson

**Affiliations:** a Research Scientist, Department of Sexual and Reproductive Health and Research, World Health Organization/Human Reproduction Programme, Geneva, Switzerland; b Independent Expert, Geneva.; c Technical Specialist, Sexual and Reproductive Health Division, Technical Branch, United Nations Population Fund (UNFPA), New York, NY, USA; d Consultant, Department of Sexual and Reproductive Health and Research, World Health Organization/Human Reproduction Programme, Geneva, Switzerland; e Senior Country Director, Pathfinder International, Addis Ababa, Ethiopia; f Paediatrician, Mamta Health Institute for Mother and Child, New Delhi, India; g Independent Expert, Geneva, Switzerland

**Keywords:** sexual health, reproductive health, adolescent well-being, adolescent reproductive health, programmes, low- and middle-income countries, multi-burden countries

## Abstract

This commentary is in response to a paper published in the Lancet entitled: “Progress in adolescent health and well-being: tracking 12 headline indicators for 195 countries and territories, 1990-2016” (Peter Azzopardi et al, 2019). We agree with the authors' overall conclusions that although there has been progress in some health outcomes, health risks and social determinants, the situation has worsened in other areas. Other important messages emerge from studying the data with an adolescent sexual and reproductive health and rights (ASRHR) lens. First, notable – albeit uneven – progress in all the ASRHR indicators has occurred in multi-burden countries. Second, while we cannot assign a cause-effect relationship, it is reasonable to suggest that in addition to secular trends, deliberate global and national investment and action have contributed to and/or accelerated these changes. Third, progress in ASRHR in the multi-burden countries contrasts sharply with increases in rates of tobacco use, binge drinking and overweight and obesity, in all categories of countries. Based on these observations, we submit five implications for action: the adolescent health community must recognize the progress made in ASRHR; acknowledge that increasing investment and action in ASRHR has contributed to these tangible results, which has the potential to grow; build on the gains in ASRHR through concerted action and a focus on implementation science; expand the adolescent health agenda in a progressive and strategic manner; and contribute to wider efforts to respond to adolescents' health needs within the rapidly changing context of the worlds they live in.

## **Résumé**

Cet article réagit à une publication dans le Lancet intitulée « Progrès dans la santé et le bien-être des adolescents: suivi de 12 principaux indicateurs dans 195 pays et territoires, 1990–2016 (Peter Azzopardi et al, 2019). Nous sommes d’accord avec les conclusions globales des auteurs qui affirment que, si des progrès ont été accomplis dans certains résultats sanitaires, risques pour la santé et déterminants sociaux, la situation a empiré dans d’autres domaines. Néanmoins, d’autres messages importants ressortent de l’étude des données dans une optique de santé sexuelle et reproductive des adolescents (SSRA). Premièrement, des progrès notables, quoiqu’inégaux, dans tous les indicateurs de SSRA ont été enregistrés dans les pays à charges multiples de morbidité. Deuxièmement, même si nous ne pouvons pas attribuer une relation de cause à effet, il est raisonnable de suggérer qu’en plus des tendances durables, des mesures et des investissements nationaux et internationaux délibérés ont contribué à ces changements et/ou les ont accélérés. Troisièmement, les progrès de la santé sexuelle et reproductive des adolescents dans les pays à charges multiples de morbidité contrastent nettement avec l’augmentation du taux de tabagisme, de la consommation ponctuelle immodérée d’alcool ainsi que du surpoids et de l’obésité, dans toutes les catégories de pays. Sur la base de ces observations, nous avons présenté les cinq conséquences suivantes pour l’action: la communauté de santé des adolescents doit prendre acte des progrès accomplis dans le domaine de la SSRA; admettre qu’une hausse des investissements et des activités dans le domaine a contribué à ces résultats tangibles et qu’il existe un potentiel de croissance; s’appuyer sur les progrès dans la SSRA avec une action concertée et une priorité aux sciences de l’application; élargir le programme de santé des adolescents de manière progressive et stratégique; et contribuer aux efforts élargis pour répondre aux besoins de santé des adolescents dans le contexte en évolution rapide des mondes dans lesquels ils vivent.

## Resumen

Este artículo fue redactado en respuesta a un trabajo publicado en la revista *The Lancet* titulado: “Progreso en salud y bienestar de adolescentes: seguimiento de 12 indicadores principales en 195 países y territorios, 1990–2016” (Peter Azzopardi et al, 2019). Estamos de acuerdo con las conclusiones generales de los autores que indican que, aunque se ha visto progreso en algunos resultados de salud, riesgos de salud y determinantes sociales, la situación ha empeorado en otras áreas. Sin embargo, otros mensajes importantes surgen al estudiar los datos desde la perspectiva de salud sexual y reproductiva de adolescentes (SSRA). En primer lugar, progreso notable, aunque desigual, en todos los indicadores de SSRA ha ocurrido en países con altos índices de enfermedad. En segundo lugar, aunque no podemos asignar una relación causa-efecto, es razonable sugerir que además de tendencias seculares, la inversión y acción deliberadas a nivel mundial y nacional han contribuido a estos cambios y/o los han acelerado. En tercer lugar, el progreso en SSRA en países con altos índices de enfermedad contrasta sobremanera con los aumentos en las tasas de consumo de tabaco, consumo excesivo de alcohol, y sobrepeso y obesidad, en todas las categorías de los países. A raíz de estas observaciones, presentamos las siguientes cinco implicaciones para tomar acción: la comunidad de salud de adolescentes debe reconocer el progreso logrado en SSRA; reconocer que mayor inversión y acción en SSRA han contribuido a estos resultados tangibles y que esto tiene el potencial de incrementar; ampliar las ganancias en SSRA por medio de acción concertada y un enfoque en ciencias de la implementación; ampliar la agenda de salud de adolescentes de una manera progresista y estratégica; y contribuir a mayores esfuerzos por responder a las necesidades de salud de adolescentes dentro del contexto en rápida evolución del mundo en que viven.

## Introduction

We congratulate Azzopardi et al^1^ for generating and consolidating an impressive body of data (including modelled data) on 12 indicators of adolescent health and for placing them in the public arena in their recently published paper, “Progress in adolescent health and well-being: tracking 12 headline indicators for 195 countries and territories, 1990–2016”.^1^ The authors have provided data on health outcomes Group 1 Disability-Adjusted Life years [DALY] due to communicable, maternal and nutrition diseases, injuries and non-communicable diseases, Injury DALYs, Noncommunicable disease DALYs, health risks (tobacco use, binge drinking, overweight and obesity and anemia), and social determinants (completion of secondary education, not in employment, education or training, adolescent live births, child marriage, demand for modern contraception satisfied). Countries have then been placed into three categories – multi-burden countries, injury-excess countries and non-communicable disease predominant countries* – based on the authors’ epidemiological assessment.

In summary, the authors note that over the 16-year period between 1990 and 2016, the adolescent disease burden has fallen; the rates of health risks (tobacco use, binge drinking, overweight and obesity) have increased while that of anaemia has decreased slightly; enrolment in secondary education has increased but employment has not, and contraceptive uptake has improved. They stress that even where rates have improved because of increases in the proportion of the adolescent population, absolute numbers of adolescents at risk have increased, and provide estimates of anaemia to make this point. Finally, they note that health, education and legal systems have failed to keep pace with adolescents’ needs and demographic changes, and gender inequality continues to be a powerful driver of poor health and well-being.

We have studied the data and the authors’ interpretations and recommendations. We agree with the authors’ overall conclusion that although there has been progress in some health outcomes, health risks and social determinants, the situation has worsened in other areas. We also agree that progress has been overshadowed by powerful demographic changes, with which national health, education, social welfare and other systems are struggling to keep pace, and that much more needs to be done to ensure that all adolescents grow and develop in good health and to their full potential.

Having said that, using an adolescent sexual and reproductive health and rights (ASRHR) lens, we see important and clear trends that are worthy of comment. In this paper, we have tabled three such observations, drawing from the data in the paper and its annexes and, in some areas, using data from other sources. We focus our critique mainly on multi-burden countries, with some comparisons to countries in the other two categories when discussing variations in adolescent health risks (tobacco, binge drinking, overweight and obesity). We have then set out five implications of our observations.

## Observations

First, important – albeit uneven – progress has occurred in multi-burden countries, in every one of the ASRHR indicators (maternal health, adolescent live births, child marriage, demand for modern contraception). In the enormous amount of detail that Azzopardi et al^1^ provide, this important point may be missed. Box 1 highlights this progress in terms of health outcomes and social determinants – some of the indicators included by Azzopardi et al are determinants of ASRHR and have been included to provide a context for the progress in ASRHR. The data discussed are derived from Azzopardi et al’s analyses and have been complemented with additional data where key indicators were lacking. The additional data provided relate to the same time period as those reported on by Azzopardi et al.

Box 1. **Changes in health outcomes and social determinants that relate to adolescent sexual and reproductive health in multi-burden countries**

**Health outcomes**

Group 1 DALYS (communicable, maternal and nutritional diseases) have decreased in multi-burden countries as a whole between 1990 and 2016, with the biggest declines occurring in countries such as Rwanda (−2.77%), Bangladesh (−2.64%), Laos (−2.62%), Nepal (−2.62%) and Myanmar (−2.56%) and the smallest decline occurring in Sierra Leone (−0.16%). An important contributor to this decline is a reduction in new HIV infections among adolescents aged 10–19 years. For example, UNAIDS’ 2017 estimates point to decreases in HIV among adolescents in this age group in multi-burden countries, between 1990 and 2017, such as Zimbabwe by 23,900, Zambia by 7400, and Uganda by 5600.^2^ Similarly, there have been declines in the maternal mortality ratio (MMR) (a component of Group 1 DALYs that constitute the multi-burden category). Unfortunately, the available data on maternal mortality for adolescents does not allow age-disaggregation of MMR since age-specific data are not gathered/analysed and reported. However, overall, MMR decreased by 38% between 2000 and 2017, with an average annual decline of 3.6% in least developed countries, which include most of the multi-burden countries.^3^ This is more than double the average annual decline of 1.3% between 1990 and 2000.^4^

**Social determinants**

Notable improvements in social determinants have also occurred in multi-burden countries between 1990 and 2016, such as an increase in completion of secondary education (14.9%), a decrease in adolescent live births (1.7%) and an increase in demand for modern contraception satisfied (2.1%), although there are large variations in these improvements between countries: increases in secondary education among females ranged from 0.01% in Lesotho to 49.3% in Yemen and increases in modern contraception satisfied ranged from 0.36% in Indonesia to 42.8% in Burundi. Although Azzopardi et al do not present trend data on child marriage, a 2018 UNICEF report indicates that the proportion of young women (20-24 years) who were married as children decreased by 15% in the last 10 years.^5^ One notable example is Ethiopia, where the prevalence of child marriage dropped by a third during the decade.^5^ Survey data also show a significant decrease in child marriages in India (19.2%) between 2005 and 2015,^6,7^ as well as modest decreases in Sierra Leone (9.0%) between 2008-2013,^8,9^ Malawi (6.8%) between 2004 and 2015,^10,11^ Rwanda (6.5%) between 2005-2014,^12,13^ and Burkina Faso (0.3%) between 2003 and 2010.^14,15^ Appendix 7 in the Azzopardi et al (2019) paper also shows that while levels of adolescent child bearing have declined overall, there is varying progress across the multi-burden countries, with the biggest declines occurring in Bhutan (3.60%), India (3.16%) and Kiribati (3.08%), and the smallest decline occurring in Cote d’Ivoire (0.04%).

Azzopardi et al highlight the impact of demographic changes on the progress that is being made. For example, although the rates of secondary school completion have increased between 1990 and 2016, there has been an even larger increase in the denominator: the population of adolescents who currently need secondary school education (and those who will need it in the future). However, this should not be seen as a failure of adolescent programmes. It means that while goals have been scored during the period, the goal posts have now been shifted. Fortunately, it also means that the adolescent health and development community can build on its successes as it responds to a challenge which has grown in size. This is analogous, in some respects, to WHO’s Three by Five Initiative, which was launched in 2003 with the aim of having three million people living with HIV on antiretroviral treatment by the year 2005. By 2005, although substantial progress had been made, the target had not been reached and, in addition, the number of people requiring antiretroviral treatment had increased substantively. However, the ground was set for rapid and substantial antiretroviral scale-up in low- and middle-income countries.^16^

Second, it is plausible that the significant resources that have supported the implementation of a range of evidence-based interventions over the past several decades, acting at different levels, have had a positive impact on ASRHR outcomes, even if this is difficult to prove scientifically because of the complex web of causation underlying ASRHR outcomes (e.g. adolescent pregnancy). For example, child marriage rates have declined in Ethiopia and India (as discussed later) but it is difficult to pin down which specific interventions contributed to this. Given existing evidence-based interventions (cited in WHO’s recommendations on adolescent sexual and reproductive health and rights,^17^ which draw from research studies and evaluations published in the international peer-reviewed literature, for example, in improving school enrolment and retention or in formulating laws to ban child marriage and change social norms), it is plausible that these changes have occurred as a result of the collective efforts of many players from the local to the international levels.

There are many examples of investments and actions in support of ASRHR over the past decades, often with a specific focus on adolescent girls:

HIV:

Recognising that adolescents were the only age group with a rising mortality as the Millennium Development Goals target date approached,^18^ UNAIDS/UNICEF launched the *All In to end adolescent AIDS (All In)*;^19^ the Global Fund to Fight AIDS, Tuberculosis and Malaria (GFATM) launched an initiative to prevent HIV in adolescent girls and young women;^20^ and PEPFAR launched the *Dreams Initiative* to reduce HIV/AIDS amongst adolescent girls.^21^

Child Marriage:

The collective efforts of non-government organisations such as PLAN International and the Population Council, United Nations agencies such as UNFPA and UNICEF, civil society organisations such as the Elders and GirlsNotBrides, and the Canadian and British governmental development agencies, have spearheaded a global movement to end child marriage. This, in turn, has led to enormous investment, including the establishment of a joint UNFPA-UNICEF-UN Women Global Programme to Accelerate Action to End to Child Marriage. In many countries this added to – and added visibility to – the work that indigenous civil society bodies and NGOs had been doing for many years to end this harmful traditional practice. ^22^

Secondary Education:

There has been a huge push to step up efforts to promote secondary school entry and completion, both as an end in itself and as a means to prevent child marriage and adolescent pregnancy. The Global Partnership on Education For All is one of a number of efforts in this area.^23^ These efforts have contributed to concerted national action to expand countries’ focus from universal primary education to secondary school entry and retention.^23^ A number of countries have put in place financial incentive programmes to encourage and support needy families to keep their girls in school. In India, initiatives such as the provision of free education and learning materials for girls, the mid-day meal scheme, the *Beti Bachao Beti Padhao* (Save Girl Child and Educate Girl Child) initiative led by the Ministries of Health and Family Welfare, Women and Child Development, and Human Resource Development, aim to address the declining child sex ratio and related areas of girls’ empowerment. Bangladesh’s progress has been extensively documented and evaluated. An evaluation published in 2009 noted that while causality is difficult to establish, data suggest that their stipend programme has contributed to the rise in enrolment of girls in secondary schools.^24^ A more recent World Bank report notes that secondary school enrolment for girls jumped from 39% in 1998 to 67% in 2017 in Bangladesh.^25^ Likewise, Yemen implemented a National Strategy on Basic Education to increase the enrolment of children, particularly girls, in education, as a national social and developmental priority. This contributed to a striking increase in secondary education (49.32%) among females between 1990 and 2016.^26^ Sadly, it is very likely that these gains have been lost because of the war in the country/region.

Adolescent contraception:

Since the International Conference on Population and Development (ICPD), a number of organisations, both international, such as the International Planned Parenthood Federation, and indigenous, have been advocating for greater attention to adolescent contraception. Over the years, this movement has gathered steam. Following its midpoint review, FP2020, a global partnership to empower women and girls by investing in rights-based family planning, identified adolescent contraception as one of its top priorities.^27^ FP2020 supported countries to make commitments on adolescent contraception at the Family Planning Summit (London, July 2012); to translate these broad endorsements into specific policy, programme, and financial commitments; and to act on them.^28^ The 2019 ICPD plus 25 Summit in Nairobi reiterated these sentiments with over 1000 commitments made by various stakeholders on programme and policy action, including financial resources.^29^

Third, progress in ASRHR in the multi-burden countries contrasts sharply with the lack of progress in other areas of health and well-being, with increases in the rates of tobacco use, binge drinking and overweight and obesity being of notable concern. In countries in the injury-excess and non-communicable disease categories, while tobacco use has declined, there has been no progress in binge drinking and in overweight and obesity. England is a case in point. The country succeeded in reducing teenage pregnancy through a textbook example of a well-designed and well-executed public health programme.^30^ As per the estimates published by Azzopardi et al, the rate of early childbearing in England declined but the rates of binge drinking and of overweight and obesity increased. This highlights two issues. Firstly, despite the availability of sizeable human and financial resources, a high-income country such as England has not yet been able to get a handle on these health risks, a trend that is also observed in other high-income NCD-predominant countries such as Singapore and Austria. And secondly, the fact that a country has been able to address effectively a sexual and reproductive health outcome or social determinant, does not necessarily mean that it will be able to address other health outcomes, health risks, and social determinants. This demonstrates the complexity in addressing these issues, even for countries with substantial human and financial resources. The adolescent groups most affected by these conditions, the drivers of these problems, the actions to be taken, and the individuals and organisations that need to carry them out, are often not the same.

## Implications for action

### Recognise that progress has been made in ASRHR

For many years, the global community has lamented the lack of progress in ASRHR.^31^ It is therefore important that we celebrate the progress that *has* been achieved in multi-burden countries. Even though the progress has been limited and uneven, we are now able to demonstrate progress among adolescents across a range of ASRHR indicators in a number of multi-burden countries. Evidence from successful national-level adolescent pregnancy prevention programmes led by governments in middle-income countries, such as Chile, and in low-income countries, such as Ethiopia, give a clear signal that progress on ASRHR is possible at the national level, beyond small-scale/time-limited projects, even in low resource settings.^32^ However, while we recognise the progress that has been made, we must acknowledge that there is an enormous unfinished agenda in ASRHR.

### Acknowledge that the increasing investment and action in ASRHR has contributed to these tangible results, and that this has the potential to grow

We must recognise that the improvements in ASRHR outcomes and in the underlying social determinants did not happen by chance. As illustrated above, it is plausible that these improvements are due to deliberate action at national and local levels, accompanied by increased investments in ASRHR. While it is true, as Li et al^33^ have shown, that adolescent health gets only a small piece of the global development cake,^33^ it is also true that there is more funding for specific areas of work on ASRHR than there has ever been, notably for preventing HIV, preventing child marriage, and preventing unintended/unwanted pregnancy. Here are three notable examples of this increased funding. First, PEPFAR has invested US$ 800 million in its DREAMS Project in 10 Eastern and Southern African countries between 2016 and 2020.^34^ Secondly, the Global Programme to Accelerate Action to End to Child Marriage spent nearly US$ 25 million in supporting 12 countries in 2017.^22^ Thirdly, the Global Financing Facility (GFF) is stepping up its investment in adolescent health in countries with the highest need – priority setting that steers resources to support governments by strengthening country ownership and equity in reproductive, maternal, newborn, child and adolescent health and nutrition (RMNCAH-N) outcomes.^35^ For example, one of the funders of the Health Sector Support Project in Bangladesh is financed by US$ 15 million from the GFF, which supports the implementation of an Essential Service Package in regions that are lagging behind, to deliver services at scale with sustained impact.^35^

The global adolescent health community has frequently bemoaned the lack of investment in adolescent health.^28,36^ However, thanks to the efforts of ASRHR advocates over many years, there are now growing resources to support ASRHR programming. The challenge facing countries, and those organisations that support them, is how to put these resources to the best possible use, and to demonstrate results. This will help grow the investments both for ASRHR and for the wider area of adolescent health. It is noteworthy that while much of the investment is from external sources, there are initial examples of domestic investments as well – a key feature contributing to the success of the Chilean and Ethiopian examples presented above was a substantive indigenous investment.^32^ Another example is India’s National Adolescent Health Programme, which is funded almost exclusively from indigenous sources.^37^

### Build on the gains in ASRHR through concerted action and a focus on implementation science

Multi-burden countries can build on their initial successes in ASRHR by sharpening and reinforcing their efforts. The Lancet Commission for adolescent health and well-being has called for no country to remain in the multi-burden group by 2030; for prevention priorities to include SRH, under-nutrition and infectious diseases including HIV; and for policy measures to include injury prevention.^38^ There is a sound public health and human rights basis for this prioritisation; there is also a practical basis for it. First, as Temin and Levin^39^ have stressed, addressing SRHR is an essential step to addressing adolescent health.^39^ For example, preventing child marriage, which almost always leads to school dropout and early childbearing, provides one of the foundations that enable adolescent girls to grow and develop to their full potential. Second, as discussed earlier, there is a momentum that could be built upon: countries have committed themselves to addressing ASRHR, donor support is available and growing, there is expertise in governments and in the NGO community, and there is emerging evidence from the field that progress is possible and tangible results are achievable.

The field of adolescent health has focused for much of the last twenty years on answering and communicating clear messages to two questions: why invest in adolescents (i.e. what is the evidence on the scale of the need/problem, the benefits in meeting the need/addressing the problem, and the costs of not doing so); and what to invest in (i.e. evidence on the determinants of adolescents’ needs/problems, and evidence on the effectiveness of interventions to address these determinants). To consolidate and expand progress on ASRHR, the emphasis now needs to shift to questions on *how* to deliver interventions proven to be efficacious in research studies and pilot projects, at scale with quality and equity in “real world” settings with different social, economic and cultural contexts, and how much this will cost.^40^ Post-project evaluations and implementation research are beginning to provide answers to the programmatic challenges of doing this, such as effectively delivering comprehensive sexuality education, stimulating and sustaining multi-sectoral coordination and collaboration, and overcoming health worker bias.^41–43^ The strong commitment to ASRHR and the availability of resources provides an unprecedented opportunity to push ahead with strong implementation, tied in closely to implementation research.^44^

### Expand the adolescent health agenda in a progressive and strategic manner

The call by Azzopardi et al^1^ that there are many important areas to address beyond sexual and reproductive health, such as non-communicable diseases caused by tobacco, binge drinking and overweight and obesity, is clearly supported by the epidemiological data presented. A growing number of multi-burden countries are beginning to do this. There are good reasons to move ahead in a phased manner beyond the sexual and reproductive health field, taking practical considerations into account in addition to demography and epidemiology. First, given the limited human and financial resources available in most places, the agenda could be expanded in a progressive manner. This can build on what is already being done; for example, health workers providing adolescents with contraceptive information and services could be trained and supported to use their counselling and communication skills to begin to address mental health problems as well. Second, the scale-up that is occurring in the ASRHR field drew upon years of investment in building community understanding and support, in establishing a sound evidence base, and in developing implementation experience. Investing time and effort in a learning agenda in areas that even high-income countries are struggling to address effectively, such as preventing substance abuse and overweight/obesity, can prepare countries for scale-up in the future. Third, in a context of growing conservatism, decision-makers in countries may be more comfortable pressing ahead with the non-controversial interventions included in the “essential package of age-appropriate and developmentally primed interventions for school-age children and older adolescents”, outlined by Bundy et al,^45^ such as iron folate supplementation, oral health promotion, and visual acuity screening, and set aside the more sensitive interventions included in the essential package such as providing comprehensive sexuality education and establishing linkages to sexual and reproductive health services.^45^

Ethiopia and India are both cases in point. In the Millennium Development Goals era, both countries had national adolescent reproductive health strategies, aligned to their respective countries’ commitments to reduce maternal and childhood mortality, and to prevent HIV transmission and HIV-related mortality and morbidity. In 2005, India’s Ministry of Health and Family Welfare (MOHFW) put in place the National Adolescent Reproductive Health Strategy and the Ministry of Health in Ethiopia developed the National Adolescent and Youth Reproductive Strategy in 2007.^46,47^ National government-led efforts, complemented by the efforts of international and indigenous NGOs and aided by economic progress in both countries, contributed to significant decreases in levels of child marriage and in adolescent childbearing (the latter more in India than in Ethiopia). The child marriage rate in India declined from 50% to 25% between 1993 and 2016 and the adolescent fertility rate in the country declined from 116 to 51 births per 1000 women aged 15–19 in the same time period.^6,48^ In Ethiopia on the other hand, while the child marriage rate declined by 9% between 2000 and 2016, the adolescent fertility rate only slightly declined from 110 to 80 births per 1000 women aged 15–19.^49,50^ The increase in contraceptive use and especially post-partum contraception in the country meant that child bearing continued to occur in adolescence but did so later.^30,50,51^

Towards the end of the Millennium Development Goals era, both countries launched broader national adolescent health strategies. In 2015 the MOHFW, India, launched the National Adolescent Health Programme (*Rashtriya Kishore Swasthya Karyakram*) with six priority areas: nutrition, sexual and reproductive health, mental health, injury and violence, substance abuse and non-communicable diseases.^37^ The Ministry of Health, Ethiopia, similarly introduced a National Adolescent and Youth Health Strategy in 2016, addressing sexual and reproductive health, HIV, sexually transmitted infections, nutrition, substance abuse, mental health, non-communicable diseases, gender-based violence and injuries.^52^ In both countries, there was clearly a sound epidemiological rationale for this move from adolescent sexual and reproductive health (ASRH) to a broader focus. However, the rapid increase in scope without effective mechanisms to translate the plans into action has meant that implementation has been patchy. At the subnational level, the focus and clarity of the adolescent reproductive health strategies have been replaced with a dilution of focus and lack of direction on the way forward – ongoing work on ASRH has suffered while work on an expanded set of areas has made limited and uneven progress.^53,54^

Defining priorities requires an understanding of the many issues that need to be addressed, the availability of evidence-based interventions and the potential for action, for example, local expectations and capacity. There are important precedents in other areas. For example, even though many things needed to be done to improve child health, there was a deliberate decision to focus on child survival: to reduce childhood mortality from commonly occurring conditions using interventions that were proven to be effective and feasible to deliver in resource-constrained settings. Only when this was on track was the agenda broadened, in a steady and progressive manner, to include other important issues, such as early childhood development.^55,56^

### Contribute to wider efforts to address adolescents’ rapidly changing contexts, that have an impact on their health and development

Finally, while there have been some improvements, especially in relation to the ASRHR indicators, the demographic changes that have occurred during the same period mean that these improvements have been outpaced by population growth. It is clearly very important to be aware of the powerful contextual changes influencing adolescent health and well-being. In addition to demographic changes, the Guttmacher–Lancet Commission on Sexual and Reproductive Health and Rights has discussed the influence of displacement and conflict, climate change and social and economic changes such as urbanisation.^36^ These changes do not affect adolescent health alone; they have implications across the life-course and in virtually every other field of health and development, including food security, water and sanitation, housing, education and employment.^56^ Neither can they be addressed by those working on adolescent health or by piecemeal efforts alone. They call for whole-of-government approaches at the country level, engaging a range of sectors and actors, and firmly grounded in well-coordinated inter-country efforts. Globally, regionally, and nationally, those working on adolescent health should be fully aware of and actively engage in such efforts, so as to ensure that these wider efforts take into account the special needs and perspectives of adolescents.

## Conclusion

The aim of our commentary is to (i) tease out and direct a spotlight on selected findings in Azzopardi et al’s paper, in particular, the levels and trends of the ASRHR indicators used in their analyses, with a focus on multi-burden countries; (ii) highlight the progress that has been achieved in these indicators, albeit uneven and often small/slow; (iii) link these changes to the investments and efforts made at global and national levels; and (iv) point to the implications of this for strengthening ASRHR programmes while gradually broadening the adolescent health agenda to include other priority health problems.

In order to do this, it will be important to recognise and learn from the preliminary successes achieved in the ASRHR field, and to take advantage of the growing investments in this field, and the growing commitment of global, regional and national players to address the ASRHR unfinished business. The progress made in ASRHR represents a never-before opportunity both to build on and to expand the gains in ASRHR and, in doing so, contribute to improving adolescent health and development more widely.^57^

## Footnotes

* The authors have categorised countries as multi-burden if adolescents (aged 10–24 years, both sexes combined) had a burden of communicable, maternal, and nutritional conditions (group 1 DALYs) of 2500 DALYs or more per 100,000 adolescents.
